# Gomisin M2 Inhibits Mast Cell-Mediated Allergic Inflammation *via* Attenuation of FcεRI-Mediated Lyn and Fyn Activation and Intracellular Calcium Levels

**DOI:** 10.3389/fphar.2019.00869

**Published:** 2019-08-02

**Authors:** Hima Dhakal, Soyoung Lee, Eun-Nam Kim, Jin Kyeong Choi, Min-Jong Kim, Jinjoo Kang, Young-Ae Choi, Moon-Chang Baek, Byungheon Lee, Hyun-Shik Lee, Tae-Yong Shin, Gil-Saeng Jeong, Sang-Hyun Kim

**Affiliations:** ^1^Cell & Matrix Research Institute, School of Medicine, Kyungpook National University, Daegu, South Korea; ^2^Department of Pharmacology, School of Medicine, Kyungpook National University, Daegu, South Korea; ^3^Immunoregulatory Materials Research Center, Korea Research Institute of Bioscience and Biotechnology, Jeongeup, South Korea; ^4^College of Pharmacy, Keimyung University, Daegu, South Korea; ^5^Molecular Immunology Section, Laboratory of Immunology, National Eye Institute, National Institutes of Health, Bethesda, MD, United States; ^6^Department of Molecular Medicine, School of Medicine, Kyungpook National University, Daegu, South Korea; ^7^Department of Biochemistry and Cell Biology, School of Medicine, Kyungpook National University, Daegu, South Korea; ^8^School of Life Sciences, Kyungpook National University, Daegu, South Korea; ^9^College of Pharmacy, Woosuk University, Jeonju, South Korea

**Keywords:** mast cells, allergic inflammation, anaphylaxis, calcium, Gomisin M2

## Abstract

Mast cells are effector cells that induce allergic inflammation by secreting inflammatory mediators. Gomisin M2 (G.M2) is a lignan isolated from *Schisandra chinensis* (Turcz). Baill. exhibiting anti-cancer activities. We aimed to investigate the anti-allergic effects and the underlying mechanism of G.M2 in mast cell–mediated allergic inflammation. For the *in vitro* study, we used mouse bone marrow–derived mast cells, RBL-2H3, and rat peritoneal mast cells. G.M2 inhibited mast cell degranulation upon immunoglobulin E (IgE) stimulation by suppressing the intracellular calcium. In addition, G.M2 inhibited the secretion of pro-inflammatory cytokines. These inhibitory effects were dependent on the suppression of FcεRI-mediated activation of signaling molecules. To confirm the anti-allergic effects of G.M2 *in vivo*, IgE-mediated passive cutaneous anaphylaxis (PCA) and ovalbumin-induced active systemic anaphylaxis (ASA) models were utilized. Oral administration of G.M2 suppressed the PCA reactions in a dose-dependent manner. In addition, G.M2 reduced the ASA reactions, including hypothermia, histamine, interleukin-4, and IgE production. In conclusion, G.M2 exhibits anti-allergic effects through suppression of the Lyn and Fyn pathways in mast cells. According to these findings, we suggest that G.M2 has potential as a therapeutic agent for the treatment of allergic inflammatory diseases *via* suppression of mast cell activation.

## Introduction

Mast cells are primary immune cells with pivotal roles in allergic inflammatory reactions (Wernersson and Pejler, 2014). Mast cells secrete a wide range of biological mediators, which collectively account for the pathology of allergic inflammation (Theoharides et al., 2012). These include preformed secretory granules (containing histamine and proteases) as well as cytokines, chemokines, and growth factors, which are synthesized upon mast cell activation (Espinosa and Valitutti, 2018). Among these, histamine acts as an important mediator of allergic inflammation by stimulating vasodilation, vascular permeability, hypothermia, and recruitment of leukocytes (Galli and Tsai, 2012). Furthermore, mast cells secrete pro-inflammatory cytokines contributing the progression of allergic inflammatory reactions (Metcalfe et al., 2009; Theoharides et al., 2012).

Mast cell activation is triggered by cross-linking of membrane-bound immunoglobulin E (IgE) with FcεRI, the high-affinity IgE receptor (Gilfillan and Tkaczyk, 2006). Aggregation of FcεRI is associated with activation of the Src family protein-tyrosine kinase (PTK), Lyn, which subsequently phosphorylates Syk (Gilfillan and Rivera, 2009). FcεRI-mediated signaling also activates another PTK, Fyn, which enhances Syk activation. This is followed by activation of Grb2-associated binding protein 2 and phosphoinositide 3-kinase (PI3K) (Gilfillan and Tkaczyk, 2006). Therefore, both PTKs are important for activation of various signaling pathways, including the PI3K, phospholipase C (PLC)γ, Akt, and nuclear factor (NF)-κB pathways. These signaling events lead to the initiation of inflammatory responses *via* intracellular calcium release, degranulation, and cytokine secretion (Siraganian et al., 2010).

Intracellular calcium release leads to mast cell degranulation and cytokine production in allergic inflammation (Cohen et al., 2015). FcεRI-mediated activation of PLCγ generates inositol triphosphate (IP3) and diacylglycerol (DAG), which are essential factors for intracellular calcium release and mast cell degranulation. In addition, various studies have reported that calcium releases triggers secretion of inflammatory cytokines, which is mediated by nuclear translocation of NF-κB (Je et al., 2015; Krystel-Whittemore et al., 2015; Kim et al., 2018). Nuclear translocation of NF-κB mediates the production of inflammatory cytokines, especially tumor necrosis factor (TNF)-α, interleukin (IL)-1β, and IL-6 (Gilfillan and Tkaczyk, 2006; Kim et al., 2006). Thus, inhibition of FcεRI-mediated activation of Lyn, Fyn, and intracellular calcium levels are considered as potential therapeutic strategy in mast cell–mediated allergic inflammation.

The fruit of *Schisandra chinensis* (Turcz). Baill. (*Schisandraceae*) is well-known in traditional Chinese medicine and Western herbal medicine and is widely used for the treatment of cough, asthma, diaphoresis, rheumatism, and arthritis (Lee et al., 2007; Kim et al., 2014). Several studies have demonstrated that *S. chinensis* exhibits diverse pharmacological effects, including anti-allergic, anti-inflammatory, anti-oxidant, anti-tumor, anti-viral, anti-bacterial, and hepatoprotective properties (Chae et al., 2011; Szopa et al., 2017). *S. chinensis* consists of various bioactive constituents, including lignans, triterpenoids, polysaccharides, and sterols (Opletal et al., 2004). Many active lignans have been extracted from this plant such as deoxyschisandrin, schisandrin, γ-schisandrin, and gomisin (Szopa et al., 2017). Among these, schisandrin and gomisin N have been reported to possess anti-allergic inflammatory effects on mast cells (Lee et al., 2007; Chae et al., 2011). Gomisin M2 (G.M2) is one of the active lignin components of *S. chinensis* and has shown anti-HIV properties by inhibiting the replication of H9 lymphocytes and demonstrated cytotoxicity against MCF7 and CAL27 cancer cells (Chen et al., 2006; Hou et al., 2016). In addition, G.M2 has been considered a quality marker of a Chinese herbal formulae, Shengmai San, for protection against Alzheimer’s disease (Zhang et al., 2018). Based on the anti-allergic effects of other extracts isolated from *S. chinensis*, we hypothesize that G.M2 may have anti-allergic activity in mast cell–mediated allergic inflammation.

## Materials and Methods

### Extraction, Isolation, and Identification of G.M2

Fruits of *S. chinensis* were purchased from the Yangnyeong herbal medicine market (Daegu, Republic of Korea). The specimen was identified by Prof. Jeong of the College of Pharmacy, Keimyung University, Republic of Korea, where a voucher specimen (No. KPP2018-1022) has been deposited. Fruits of *S. chinensis* (20 kg) were extracted with 95% ethanol (EtOH, 10 L) at room temperature for 5 days. The alcoholic extract was evaporated *in vacuo* to yield residue (5.7 kg), and the residue was suspended in H_2_O and successively partitioned with dichloromethane (CH_2_Cl_2_), ethyl acetate, and *n*-butanol. The CH_2_Cl_2_ extract (525 g) was subjected to silica gel column chromatography with a gradient of ether/acetone (20:1 to 1:2) to obtain five major fractions (Fr. 1–Fr. 5). Fr. 1 was subjected to Sephadex LH-20 elution with methanol/H_2_O (1:1), yielding two fractions (Fr. 1–1 and Fr. 1–2). Purification by semi-preparative high-performance liquid chromatography (HPLC) of Fr. 1–1 and Fr. 1–2 eluted with MeOH/H_2_O (10:1 to 1:1) yielded G.M_2_ (64 mg, yield 0.012%), schisandrin (902 mg, yield 0.171%), and gomisin A (752 mg, yield 0.143%) with a purity of at least 94.2%. G.M2 was identified, among the Fr. 1–2 isolates using ^1^H and ^13^C-NMR, respectively, and *via* a comparison of the generated spectral data with published data (Li et al., 2017).

G.M_2_: HRESIMS m/z: 387 [M+H]^+^; ^1^H NMR (CDCl_3_, 500MHz): δH 6.45 (H-11), 5.93 (1-H, d, OCH_2_O), 3.80 (3-H, s, OMe-12), 3.57 (3-H, s, OMe-1), 3.49 (3-H, s, OMe-13), 2.42 (1-H, dd, J = 13.4, 7.7, H-9), 2.21 (1-H, dd, J = 13.4, 1.9, H-9), 1.98 (1-H, dd, J = 13.1, 9.3, H-6), 0.93 (3-H, d, J = 7.3, H-17), and 0.70 (3-H, d, J = 7.0, H-18); ^13^C NMR(CDCl_3_, 500MHz): δC 149.6 (C-12), 147.9 (C-3), 147.5 (C-14), 139.2 (C-1), 136.9 (C-5), 134.1 (C-2), 133.6 (C-13), 133.0 (C-10), 121.0 (C-16), 117.0 (C-15), 106.1 (C-11), 103.2 (C-4), 100.7 (OCH_2_O), 59.7 (OMe-13), 58.2 (OMe-1), 55.1 (OMe-12), 40.7 (C-7), 38.4 (C-9), 35.3 (C-6), 33.2 (C-8), 21.8 (C-17), and 12.8 (C-18).

### Reagents and Cell Culture

Anti-DNP IgE, DNP-human serum albumin (HSA), *o*-phthaldialdehyde (OPA), 4-nitrophenyl N-acetyl-β-D-glucosamide, dexamethasone (Dexa), and Histodenz™ were purchased from Sigma-Aldrich (St. Louis, MO). Alum adjuvant was procured from Thermo Scientific (Waltham, MA). Mouse bone marrow–derived mast cells (mBMMCs), rat basophilic leukemia cells (RBL-2H3, ATCC: CRL-2256), and rat peritoneal mast cells (RPMCs) were cultured at 5% CO_2_ in a 37°C atmosphere in Roswell Park Memorial Institute (RPMI)-1640 medium (Hyclone, Logan, UT), Dulbecco’s Modified Eagle’s medium, and α-minimum essential medium (Gibco, Grand Island, NY), respectively. These media were supplemented by 10% heat-inactivated fetal bovine serum (Gibco), 100 µg/ml streptomycin, 250 ng/ml amphotericin, and 100 U/ml penicillin G.

### Preparation of mBMMCs

Mouse BMMCs were isolated from the femurs of male imprinting control region (ICR) mice as previously described (Kim et al., 2018). The isolated cells were grown in complete RPMI-1640 and supplemented with recombinant murine IL-3 and stem cell factor. After 4 weeks, fluorescence-activated cell sorting analysis was performed to evaluate surface markers and to confirm the mast cell purity. Cells expressing > 90% of FcεRI^+^ and c-kit^+^ were used for experiments.

### Preparation of RPMCs

RPMCs were isolated from two Sprague–Dawley (SD) rats as previously described (Kim et al., 2006). Isolated RPMCs were separated from other peritoneal cells by adding 1 ml of 0.235 g/ml Histodenz^™^ solution in cell solution followed by centrifuge filtration at 400 *g* for 15 min at room temperature. The supernatant containing other cells was discarded, and mast cells in the pellet were washed and resuspended. The purity and the viability of RPMCs were determined by toluidine blue (approximately 97%) and trypan blue (approximately 95%) staining.

### Cell Viability

Cell viability was measured using MTT Assay Kit (Welgene, Seoul, Korea) as described previously (Je et al., 2015). Briefly, mBMMCs, RBL-2H3, and RPMCs (2 × 10^4^ cells/well in a 96-well plate) were treated with G.M2 (0.01–100 µM) for 8 h, followed by incubation with MTT reagent for 2 h. The formed formazan crystals were dissolved in dimethyl sulfoxide, and the absorbance was measured at 570 nm using a plate reader (Molecular Devices).

### Histamine and β-Hexosaminidase Release

Anti-DNP IgE (50 ng/ml)–sensitized mBMMCs, RBL-2H3, and RPMCs were pre-treated with or without G.M2 (0.1–10 µM) and then challenged with DNP-HSA (100 ng/ml) for 30 min, 4 h and 2 h, respectively. To measure histamine levels in blood serum and release media, 0.1 N HCl and 60% perchloric acid were added, followed by centrifugation. The supernatant was transferred, and 5 M NaCl, *n*-butanol, and 5 N NaOH were added. The solution was then vortexed and centrifuged again. In the next step, *n*-heptane and 0.1 N HCl were added to the supernatant, which was then vortexed and centrifuged. The histamine content was measured using an OPA spectrofluorometric procedure as previously described (Je et al., 2015). The fluorescence intensity was detected at an excitation wavelength of 360 nm and an emission wavelength of 440 nm using a fluorescence plate reader (Molecular Devices). β-Hexosaminidase release in media was measured, as previously described (Kim et al., 2018). The absorbance was detected at 405 nm using a plate reader (Molecular Devices).

### Intracellular Calcium

Fluo-3/AM (Invitrogen), a fluorescent indicator, was used to measure intracellular calcium levels. mBMMCs, RBL-2H3, and RPMCs were pre-incubated with Fluo-3/AM for 1 h, treated with or without G.M2 (0.1–10 µM) for 1 h, and then challenged with DNP-HSA (100 ng/ml). The fluorescence intensity was detected at an excitation wavelength of 485 nm and an emission wavelength of 520 nm as previously described (Kim et al., 2018). Intracellular calcium levels were compared to those of untreated control cells, which were set at a value of one relative fluorescent unit.

### Quantitative Polymerase Chain Reaction (qPCR)

Anti-DNP IgE (50 ng/ml)–sensitized mBMMCs and RBL-2H3 were pre-treated with or without G.M2 (0.1–10 µM) and challenged with DNP-HSA (100 ng/ml) for 30 min and 1 h, respectively. Total cellular RNA was isolated, following the manufacturer’s protocol (RNAiso Plus Kit, Takara Bio, Shiga, Japan). Quantitative PCR was used to measure the mRNA expression of TNF-α and IL-6, as previously described (Kim et al., 2017).

### Enzyme-Linked Immunosorbent Assay (ELISA)

Anti-DNP IgE (50 ng/ml)–sensitized mBMMCs and RBL-2H3 were pre-treated with or without G.M2 for 1 h and then challenged with DNP-HSA (100 ng/ml) for 6 h. Cytokines levels were measured by ELISA Kits (BD Biosciences) according to the manufacturer’s protocol. The substrate reaction was stopped, and the absorbance was measured using a spectrophotometer at a wavelength of 450 nm, as previously described (Je et al., 2015).

### Western Blot

Anti-DNP IgE (50 ng/ml)–sensitized mBMMCs were pre-treated with or without G.M2 for 1 h and then challenged with DNP-HSA (100 ng/ml) for 5 min (Lyn, Fyn, and Syk) and 15 min (PI3K, PLCγ1, Akt, IKK α/β, IκBα, and p65 NF-κB). Total protein was extracted as described previously (Kim et al., 2018). Equal amounts of protein were electrophoresed using 7.5–10% SDS-PAGE and transferred to a nitrocellulose membrane. The membrane was incubated with specific primary antibody followed by anti-IgG horse-radish peroxidase-conjugated secondary antibody. Immunodetection was performed using an enhanced chemiluminescence detection kit (Amersham, Piscataway, NJ).

### Animals

Male ICR mice (30–35 g, 6 weeks old) and SD rats (240–280 g, 8 weeks old) were obtained from Dae-Han Experimental Animal Center (Daejeon, Korea). Throughout the study, animals (*n* = 5/cage) were provided with food and water *ad libitum* in a laminar air flow room maintained at 22 ± 2°C with a relative humidity of 55 ± 5% and 12-h light:dark cycles.

### IgE-Mediated Passive Cutaneous Anaphylaxis (PCA)

The ear of each mouse was intradermally injected with 0.5 µg/site of anti-dinitrophenyl (DNP) IgE or phosphate buffered saline (PBS) and monitored for 48 h. G.M2 (0.1–10 mg/kg) or Dexa (10 mg/kg) were then administered orally. After 1 h, mice were challenged with a mixture of DNP-HSA (1 mg/mouse) and 4% Evans blue (1:1) *via* intravenous injection into the tail vein. The mice were euthanized 30 min after intravenous injection. Ear thickness and absorbance intensity were measured as previously described (Kim et al., 2017).

### Ovalbumin (OVA)-Induced Active Systemic Anaphylaxis (ASA)

Each mouse was sensitized by repeated intraperitoneal injection of OVA mixture (100 µg of OVA and 2 mg of alum adjuvant) or PBS on days 0 and 7, as previously described (Kim et al., 2017). Subsequently, on days 9, 11, and 13, G.M2 (0.1–10 mg/kg) or Dexa (10 mg/kg) were administered orally. On day 14, mice were challenged with an intraperitoneal injection of 200 µg OVA, and rectal temperature was then monitored and recorded in at 10 min intervals for total of 90 min. Mice were euthanized after 90 min, and blood samples were collected from the abdominal artery for further experiments.

### Statistical Analysis

All statistical data were analyzed using Prism statistical software (GraphPad Software, Inc). The results are expressed as the means ± SEM of three independent *in vitro* experiments and two independent *in vivo* experiments. Treatment effects were analyzed using one-way analysis of variance followed by Tukey’s *post hoc* tests. *p* < 0.05 was considered as statistically significant.

## Results

### Effects of G.M2 on Mast Cell Degranulation

**Figure 1:**
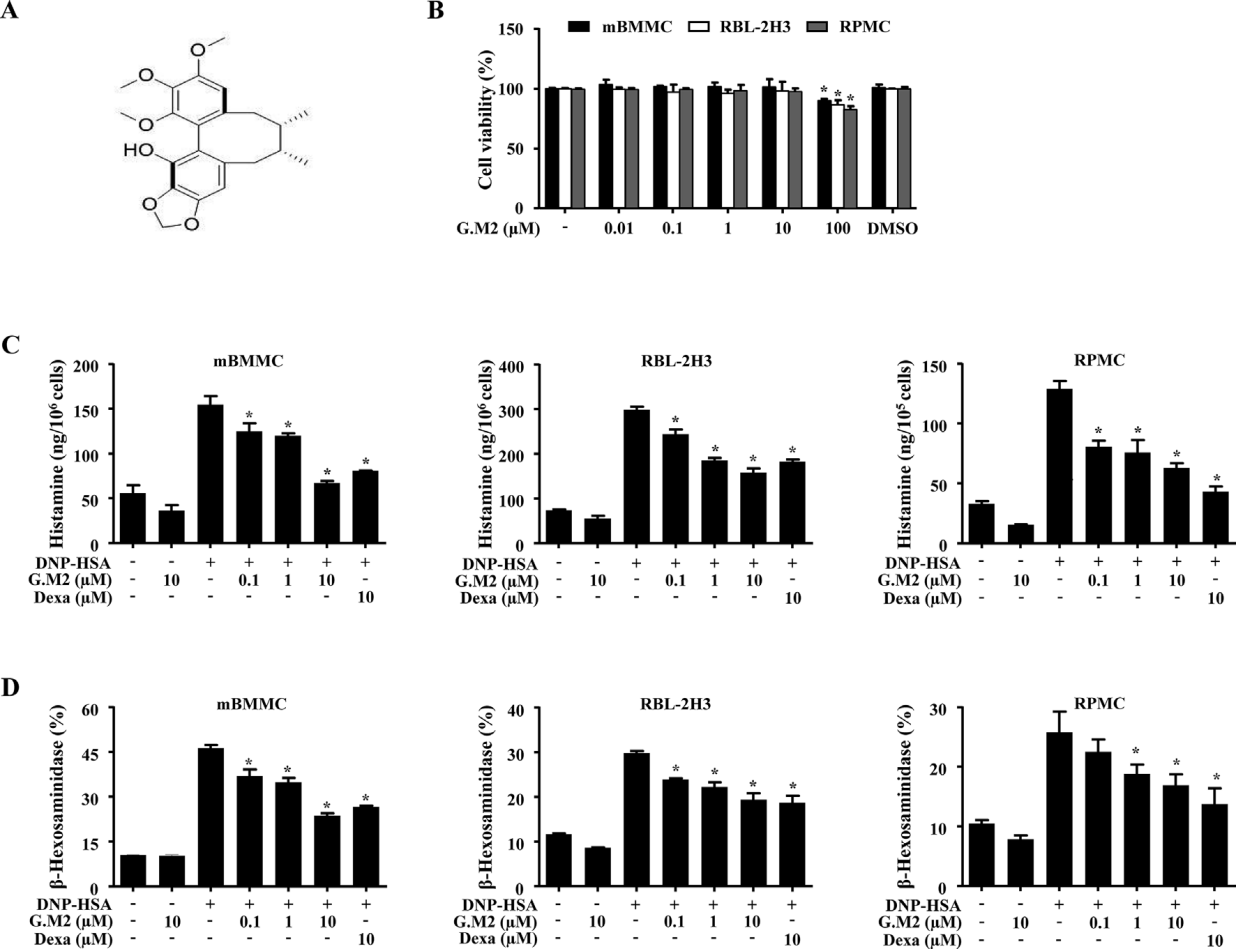
Effects of Gomisin M2 (G.M2) on mast cell degranulation. **(A)** Chemical structure of G.M2. **(B)** Mouse bone marrow derived mast cells (BMMCs), rat basophilic leukemia cells (RBL-2H3), and rat peritoneal mast cells (RPMCs) (2 × 10^4^ cells/well in a 96-well plate) were pre-treated with or without G.M2 and then incubated with MTT. The absorbance was detected using a spectrophotometer. For mast cell degranulation, anti-DNP (50 ng/ml)-sensitized mBMMCs, RBL-2H3 (5 × 10^5^ cells/well in 12-well plates), and RPMCs (3 × 10^4^ cells/well in a 24-well plate) were pre-treated with or without G.M2 or Dexa for 1 h and then challenged with DNP-HSA (100 ng/ml). **(C)** Histamine levels were detected with a fluorescence plate reader. **(D)** The level of β-hexosaminidase was measured using β-hexosaminidase substrate buffer. Each dataset presented as a graph represents the means ± SEM of three independent experiments. *p < 0.05, compared with the DNP-HSA-challenged group. Dexa: dexamethasone.

The chemical structure of G.M2 is displayed in **Figure 1A**. To determine the anti-allergic effects of G.M2, we performed a degranulation assay based on the release of histamine and β-hexosaminidase in mBMMCs. We compared the inhibitory effect of G.M2 with known anti-allergic and major components of *S. chinensis*, schisandrin, and gomisin A. As depicted in **Figure S1**, G.M2 inhibited mast cell degranulation and this inhibition was slightly greater than schisandrin and gomisin A, and the positive control drug, Dexa, at the same concentration (10 µM). We initially assessed the possible cytotoxicity of G.M2 using MTT assay. G.M2 (0.01–100 µM)-treated mBMMCs, RBL-2H3, and RPMCs were incubated for 8 h. G.M2 did not show cytotoxicity up to 10 µM (**Figure 1B**). Therefore, we decided to use G.M2 up to a concentration of 10 µM for all *in vitro* experiments. Next, we performed further experiments to elucidate the effects of G.M2 on mast cell degranulation. IgE/Ag-sensitized mBMMCs, RBL-2H3, and RPMCs were challenged with DNP-HSA. Pre-treatment with G.M2 (0.1–10 μM) markedly inhibited the release of histamine (**Figure 1C**) and β-hexosaminidase (**Figure 1D**) in a concentration-dependent manner in mBMMCs, RBL-2H3, and RPMCs compared with that in DNP-HSA-challenged cells.

### Effects of G.M2 on Intracellular Calcium Levels in Mast Cells

**Figure 2:**
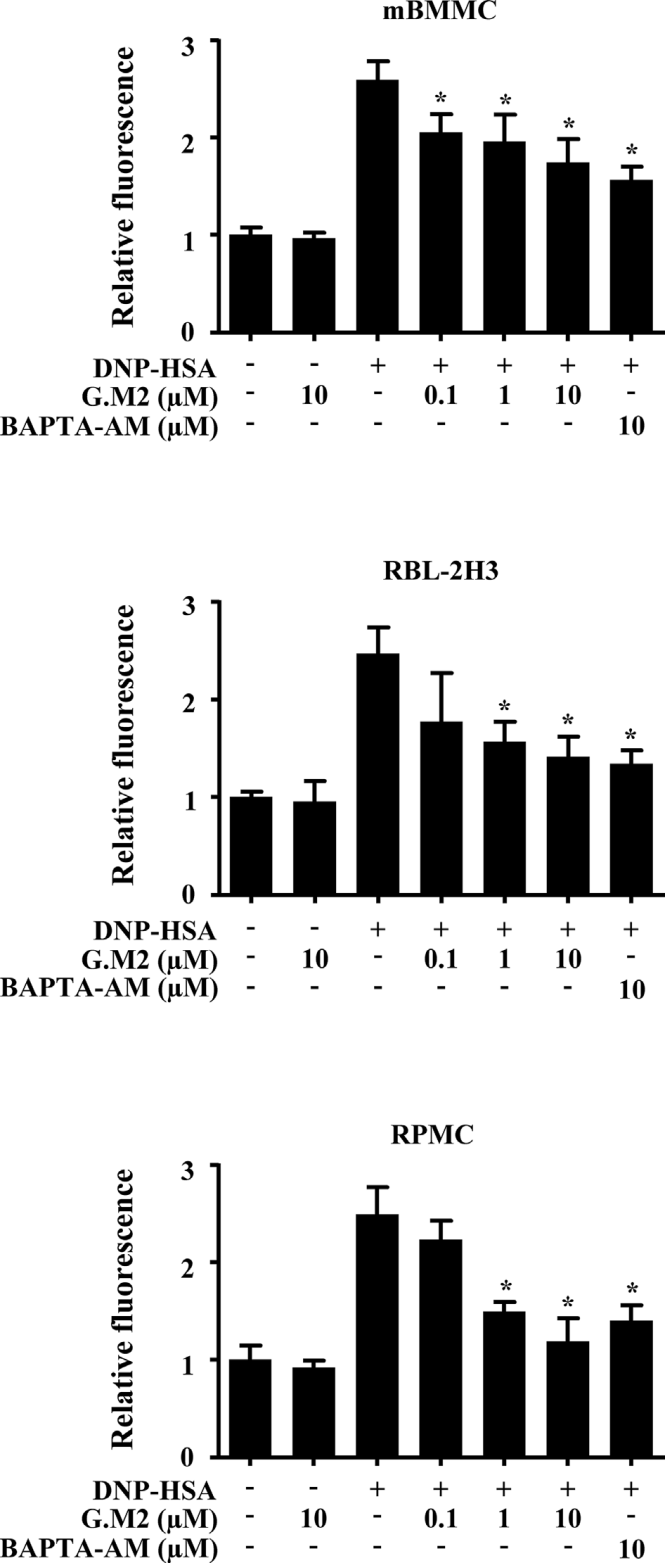
Effects of G.M2 on intracellular calcium levels in mast cells. Anti-DNP immunoglobulin E (IgE)–sensitized mBMMCs, RBL-2H3, and RPMCs (3 × 10^4^ cells/well in a 96-well plate) were pre-incubated with Fluo/3AM for 1 h, pre-treated with or without G.M2 for 1 h, and then challenged with DNP-HSA. Intracellular calcium was detected using a fluorescence plate reader. Each dataset presented as a graph represents the means ± SEM of three independent experiments. *p < 0.05, compared with the DNP-HSA-challenged group.

Increased calcium is essential for degranulation and cytokine secretion in response to mast cell stimulation (Ma and Beaven, 2011). To evaluate the mechanism by which G.M2 suppressed mast cell degranulation, we measured intracellular calcium levels. The inhibitory effects of G.M2 on intracellular calcium release were analyzed using the fluorescent indicator Fluo-3/AM. DNP-HSA-challenged mBMMCs, RBL-2H3, and RPMCs significantly elevated the intracellular calcium levels, and pre-treatment with G.M2 (0.1–10 μM) mitigated the elevation of intracellular calcium levels in a concentration-dependent manner (**Figure 2**).

### Effects of G.M2 on Expression and Release of Inflammatory Cytokines in Mast Cells

**Figure 3:**
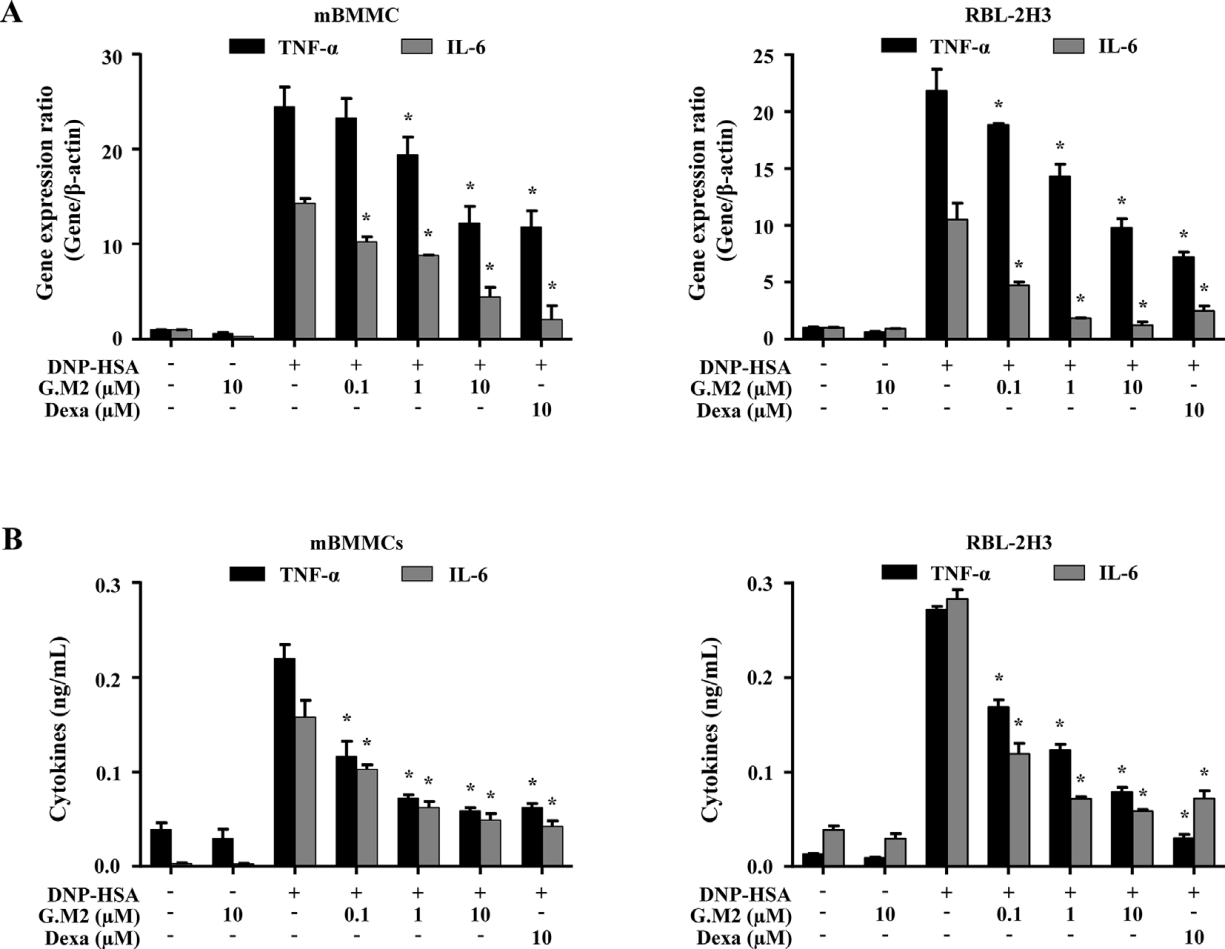
Effects of G.M2 on expression and release of inflammatory cytokines in mast cells. Anti-DNP IgE–sensitized mBMMCs and RBL-2H3 (5 × 10^5^ cells/well in 12-well plates) were pre-treated with or without G.M2 and Dexa for 1 h and then challenged with DNP-HSA (100 ng/ml). **(A)** The gene expression of inflammatory cytokines was determined by qPCR in mBMMCs and RBL-2H3. **(B)** The release of inflammatory cytokines was measured by enzyme-linked immunosorbent assay (ELISA) in mBMMCs and RBL-2H3. Each dataset presented as a graph represents the means ± SEM of three independent experiments. *p < 0.05, compared with the DNP-HSA-challenged group. Dexa, dexamethasone.

The secretion of inflammatory cytokines, such as TNF-α and IL-6 from activated mast cells, contributes to allergic inflammation (Galli et al., 2008). To evaluate the effects of G.M2 on the expression and release of pro-inflammatory cytokines in IgE-sensitized mBMMCs and RBL-2H3, qPCR and ELISA were performed, respectively. Pre-treatment with G.M2 (0.1–10 µM) significantly suppressed the gene expression (**Figure 3A**) and release of pro-inflammatory cytokines (**Figure 3B**) in a concentration-dependent manner compared with that in DNP-HSA-challenged mast cells.

### Effects of G.M2 on FcεRI-Mediated Signaling Proteins in Mast Cells

**Figure 4:**
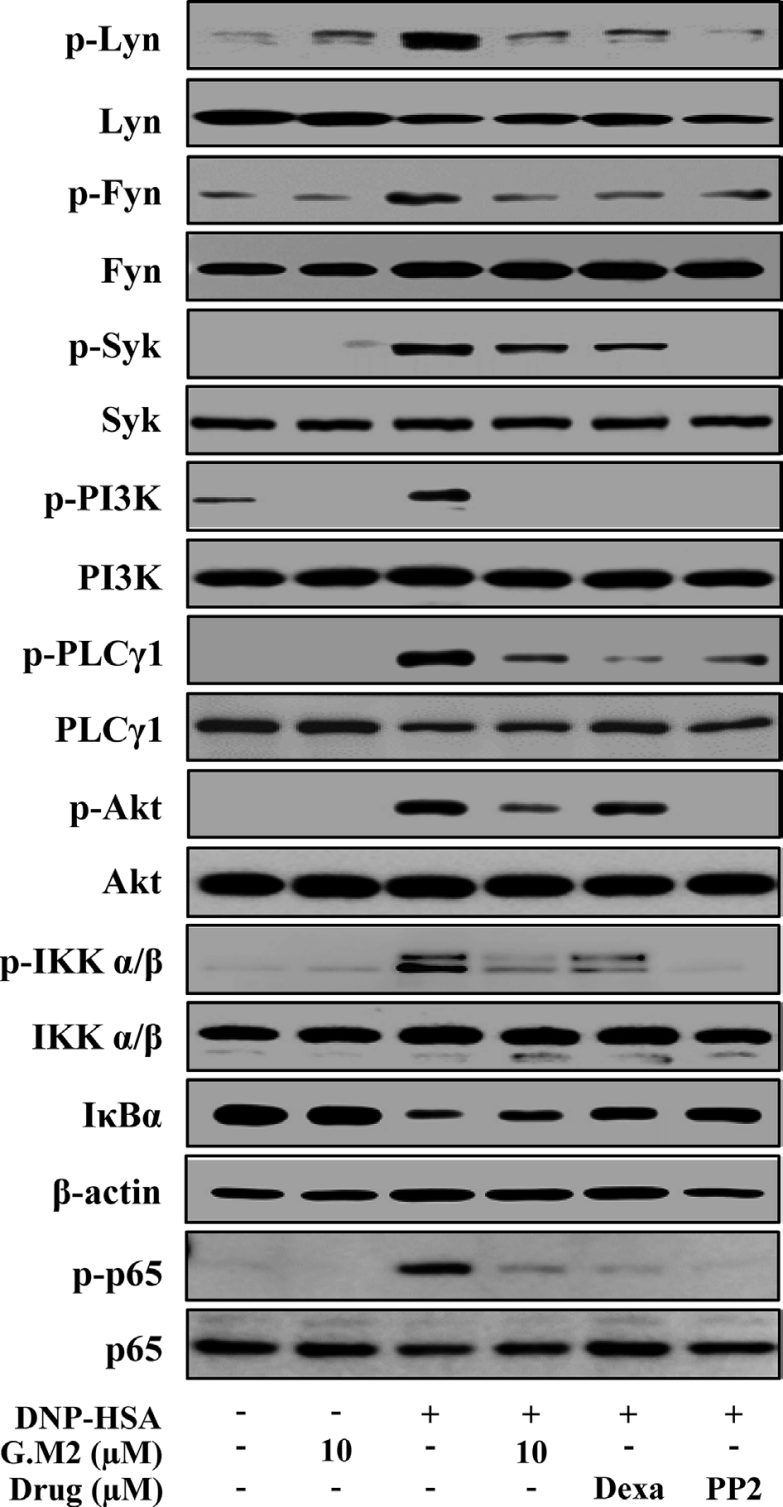
Effects of G.M2 on FcεRI-mediated signaling proteins in mast cells. Anti-DNP-sensitized mBMMCs (2 × 10^6^ cells/well in 6-well plates) were pre-treated with or without G.M2, Dexa, or PP2 for 1 h and then challenged with DNP-HSA (100 ng/ml). The activation of signaling proteins was assayed by Western blot analysis (p-: phosphorylated). Cropped blots are shown. Full-length blots are presented in **Figure S3**. The bands of β-actin and total form were used as loading controls. Each blot is a representative of three independent experiments.

Next, we evaluated the mechanism by which G.M2 inhibited secretion of inflammatory mediators that contribute to the induction and development of allergic inflammation. Therefore, we evaluated the effects of G.M2 on FcεRI-mediated signaling proteins. In mBMMCs, phosphorylations of Lyn, Fyn, Syk, PI3K, PLCγ1, IKKα/β, Akt, and p65 NF-κB were increased by DNP-HSA stimulation. However, G.M2 suppressed the phosphorylation of these proteins compared to DNP-HSA-challenged mBMMCs (**Figure 4**). In order to predict the target molecule, a well-known Src inhibitor PP2 was used as a positive control to evaluate the suppressive effects of G.M2. G.M2 showed an inhibitory effect similar to that of PP2. There is a possibility that G.M2 directly alter the expression of FcεRIα. To check this point, we determined whether G.M2 inhibits FcεRIα expression in mBMMCs and RBL-2H3 using qPCR. The result showed that G.M2 did not inhibit DNP-HSA-upregulated FcεRIα (**Figure S2**).

### Effects of G.M2 on Local and Systemic Anaphylaxis

**Figure 5:**
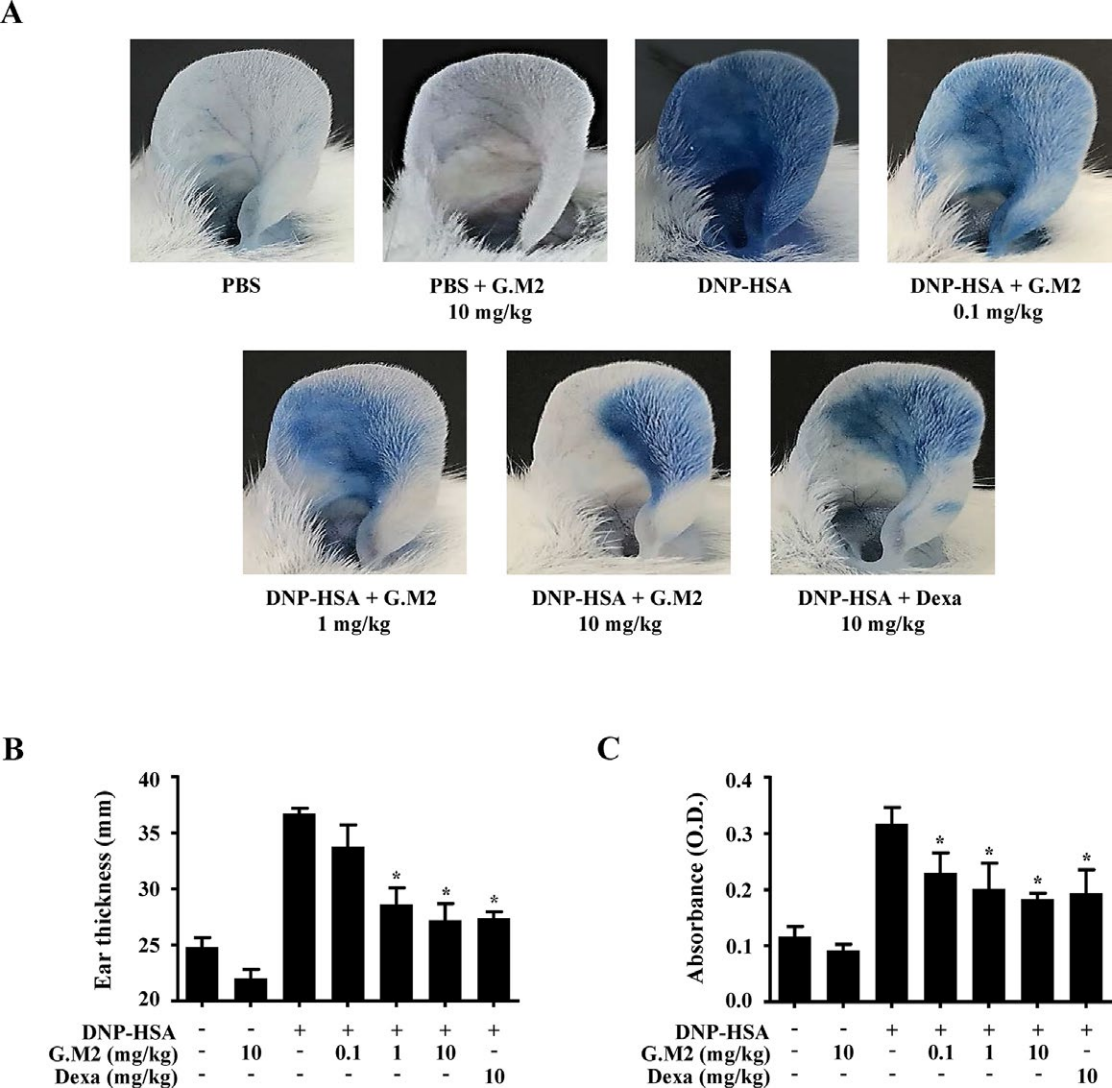
Effects of G.M2 on IgE-mediated passive cutaneous anaphylaxis (PCA). Mouse ear skin (total *n* = 35, *n* = 5/group) was sensitized with an intradermal injection of anti-DNP IgE (0.5 μg/site) or PBS for 48 h. G.M2 (0.1–10 mg/kg) and Dexa (10 mg/kg) were administered orally, 1 h before intravenous injection of DNP-HSA (1 mg/mouse) and 4% Evans blue (1:1) mixture. Thirty min later, the thickness of both ears was measured, and the ears were collected to measure pigmentation. Dye extravasation was detected using a spectrophotometer. **(A)** Representative photographs of ears. **(B)** Ear thickness. **(C)** Absorbance representing dye extravasations. Each dataset presented as a graph represents the means ± SEM (*n* = 5/group) of two independent experiments. *p < 0.05, compared with the DNP-HSA-challenged group. Dexa, dexamethasone.

Immediate-type hypersensitivity is associated with mast cell activation (Hogan et al., 2012). The *in vitro* results led us to evaluate the anti-allergic effects of G.M2 on IgE-mediated PCA and OVA-induced ASA models. These models are suitable to evaluate an anti-allergic inflammatory drug candidate *in vivo* (Hogan et al., 2012). PCA is the local anaphylaxis animal model, in which allergic reactions are induced by stimulating antigen-challenged mast cells in the dermal layer (Je et al., 2015). PCA reaction in mice was increased in both ears by DNP-HSA challenge, characterized by increased ear swelling and extravasation of Evans blue dye. These reactions were markedly inhibited by oral administration of G.M2 (0.1–10 mg/kg) in a dose-dependent manner (**Figure 5**).

**Figure 6:**
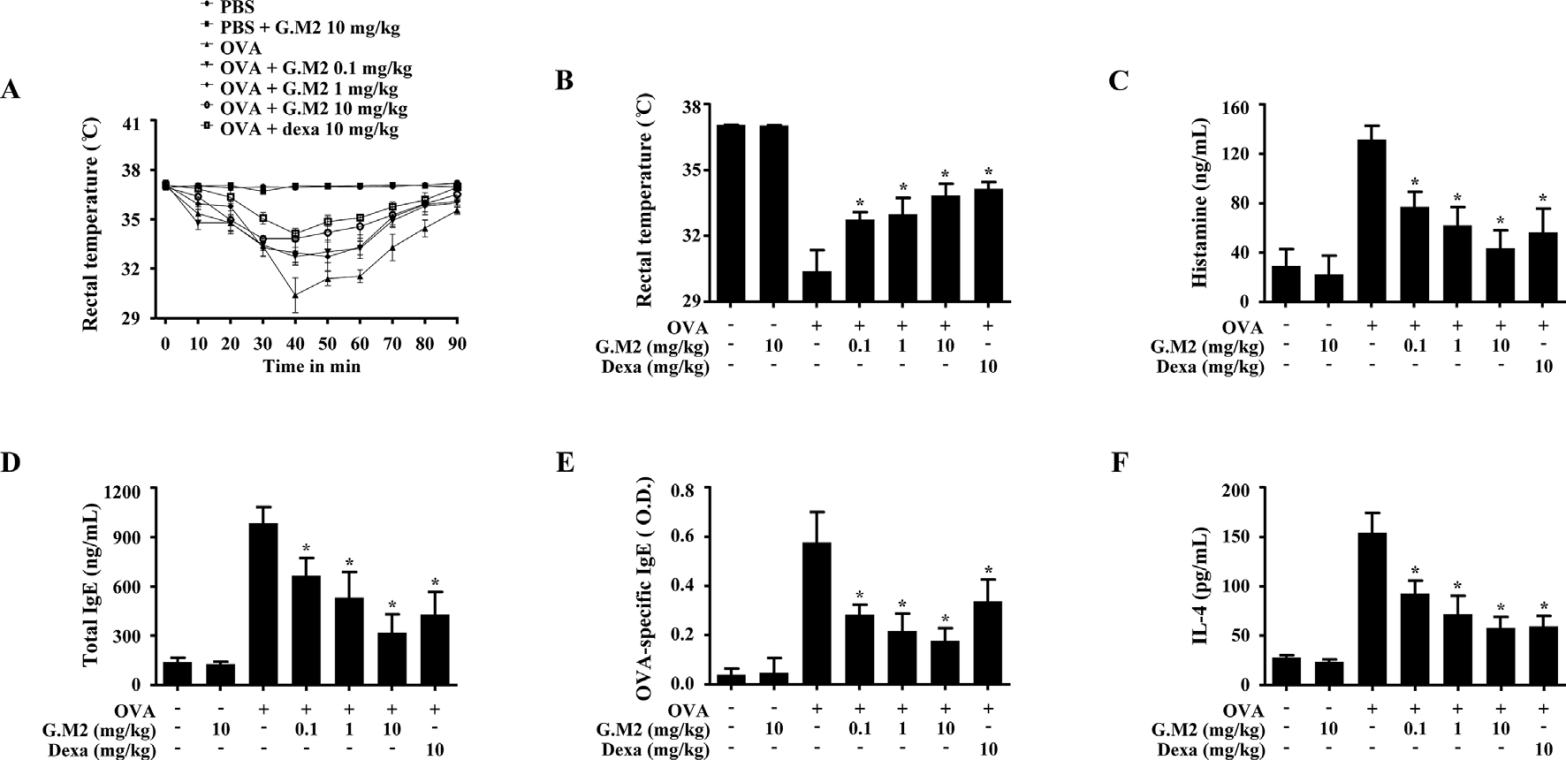
Effects of G.M2 on ovalbumin (OVA)-induced active systemic anaphylaxis (ASA). Each mouse (total *n* = 35, *n* = 5/group) was intraperitoneally injected OVA mixture (100 µg of OVA and 2 mg of alum adjuvant) or PBS on days 0 and 7. G.M2 (0.1–10 mg/kg) and Dexa (10 mg/kg) were orally administered on days 9, 11, and 13. On day 14, mice were challenged with an intraperitoneal injection of 200 µg of OVA, and rectal temperature was monitored and then recorded every 10 min for 90 min. Mice were euthanized after 90 min, and blood samples were collected from the abdominal artery. **(A)** Rectal temperature was measured every 10 min for a total of 90 min. **(B)** Rectal temperature of mice at 40 min. **(C–F)** Serum histamine, total IgE, OVA-specific IgE, and IL-4 were detected by ELISA. Each dataset presented as a graph represents the means ± SEM (*n* = 5/group) of two independent experiments. *p < 0.05, compared with the OVA-challenged group. Dexa, dexamethasone.

Systemic anaphylaxis was induced by repetitive administration of OVA and alum adjuvant followed by OVA challenge in mice. Rectal temperatures of the mice were observed for 90 min. During the period 30–60 min, mouse body temperature was decreased while serum histamine level was markedly elevated. Oral administration of G.M2 (0.1–10 mg/kg) significantly increased rectal temperature and reduced the serum histamine level in a dose-dependent manner (**Figures 6A–C**). In addition, G.M2 also suppressed the levels of serum total IgE, OVA-specific IgE, and IL-4, which were elevated after OVA challenge (**Figures 6D–F**).

## Discussion

In the present study, we used various types of murine mast cells (mBMMCs, RBL-2H3, and RPMCs) to evaluate the anti-allergic effect of G.M2, though mBMMCs were predominant cells used in our study. As non-transformed and primary cultured cells, mBMMCs are most widely used cell types for *in vitro* experiments evaluating mast cell function and molecular targets of compounds (Murakami et al., 2005). Primary cultured cells retain the functions observed *in vivo* and more closely recapitulate the endogenous drug targets physiologically and clinically (Fang, 2014). Primary cultured cells in new therapeutic drug development can be interchangeable with translational science (Eglen and Reisine, 2011). Therefore, primary cultured mBMMCs may be a suitable cell type to evaluate the anti-allergic effects and determine the molecular targets of G.M2.

The fruit of *S. chinensis* has been used as a traditional medicine in China, Korea, Japan, and Russia to treat asthma, enuresis, diabetes, dysentery, and other diseases (Liu, 1989; Panossian and Wikman, 2008). *S. chinensis* fruit extracts have been reported to possess various pharmacological properties, including anti-oxidation, anti-inflammation, and anti-allergy effects (Lee et al., 2007; Chae et al., 2011; Szopa et al., 2017). Many types of compounds were isolated from the fruits of *S. chinensis*, such as lignan, nortriterpenoid, sesquiterpene and phenolic acid; the major identified lignan constituents are schisandrin, gomisin N, and gomisin A, with reported content levels of 0.77%, 0.42%, and 0.17%, respectively (Li et al., 2013; Kim et al., 2015). Although the content of G.M2 isolated from *S. chinensis* is less, this extract confers protections from N-acetyl-*p*-aminophenol-induced liver damage, inhibits HIV replication by exhibiting potent anti-HIV activity in H9 T cell lines and reduces proliferation of cancer cells (Chen et al., 2006; Hou et al., 2016). Before elaborating the anti-allergic properties of G.M2, we initially performed degranulation assay in mBMMCs alongside the well-known anti-allergic and major components, schisandrin and gomisin A. According to our findings, G.M2 inhibited mast cell degranulation to a similar degree as the other components. Therefore, we aimed to evaluate the underlying mechanism of G.M2. Our study demonstrated that G.M2 inhibits mast cell–mediated allergic inflammation *via* inhibition of FcεRI-mediated Lyn and Fyn activation and intracellular calcium levels.

Lyn and Fyn are important intracellular mediators for the initiation of the FcεRI-mediated signaling events in mast cells (Park et al., 2018). Binding of IgE/FcεRI complexes activates both Src family tyrosine kinases Lyn and Fyn. Lyn phosphorylates the tyrosine residues of immunoreceptor tyrosine-based activation motifs (ITAMs) in the cytoplasmic regions of β and γ subunits. Phosphorylated ITAMs serve as docking sites for activation of the downstream signaling molecule Syk, which subsequently phosphorylates the transmembrane adaptor molecule linker for activation of T cells (LAT). Phosphorylated LAT directly or indirectly binds with cytosolic adaptors, such as Grb2, SLP-76, and SHC, and activates calcium signaling (Gilfillan and Tkaczyk, 2006). However, previous study showed that Lyn^-/-^ mice did not show impaired mast cell degranulation (Xiao et al., 2005; Nunes de Miranda et al., 2016). These studies suggested that another Src kinase Fyn plays a complementary role by activating various signaling pathways, including PI3K and Akt/NF-κB (Parravicini et al., 2002). In support of this hypothesis, G.M2 was found to inhibit the phosphorylation of Lyn, Fyn, Syk, IKKα/β, Akt, and NF-κB. Beyond the action, a well-known Src inhibitor PP2 was used to compare the effect of G.M2 on Src kinase, and the results showed that the inhibitory effect of G.M2 was similar to that of PP2. These results indicate that G.M2 suppresses mast cell–mediated allergic inflammation *via* inhibition of FcεRI-mediated Lyn and Fyn activation. According to these results, we propose that the molecular target of G.M2 might be Lyn and Fyn. However, considering the strong inhibition of Lyn and Fyn by G.M2, another possibility was that G.M2 inhibits FcεRI. To evaluate this hypothesis, we tested the effect of G.M2 on the expression of FcεRIα. Our results showed that G.M2 did not alter IgE-elevated FcεRIα expression. From this result, we suggest that the molecular targets of G.M2 might be Lyn and Fyn, as these are important early signaling molecules in mast cell activation.

FcεRI-mediated activation of Lyn and Fyn leads to the activation of the calcium-signaling pathway, which is critical for the calcium release (Ma and Beaven, 2011). Increased calcium level is necessary for both degranulation and cytokine secretion (Ma and Beaven, 2011). Calcium signaling is induced by receptor-mediated activation of PLCγ. Activated PLCγ cleaves phosphatidylinositol 4,5-biphosphate to generate the secondary messengers IP3 and DAG (Ma and Beaven, 2011). Binding of IP3 to its receptor induces intracellular calcium release from the endoplasmic reticulum and Golgi (Gilfillan and Tkaczyk, 2006). Moreover, a previous reported that activated PI3K increases IP3 levels, which are associated with intracellular calcium release (Ghigo et al., 2017). Interactions among these signaling molecules are required for the secretion of pro-inflammatory cytokines and mast cell degranulation, which subsequently mediate the allergic inflammation (Parravicini et al., 2002; Krystel-Whittemore et al., 2015). In our study, G.M2 inhibited phosphorylation of the calcium-signaling molecules, PLCγ, and PI3K and mitigated intracellular calcium levels. This result suggests that G.M2 inhibits mast cell activation by blocking the interaction of Lyn- and Fyn-mediated calcium signaling.

Several reports have provided evidence that intracellular calcium level is associated with pro-inflammatory cytokine secretions by mast cells, which is regulated by transcription factors (Kim et al., 2006; Bae et al., 2011; Liu et al., 2017). Many independent studies have investigated the role of NF-κB, a major transcription factor in mast cells (Bae et al., 2011; Kim et al., 2018). Activated NF-κB induces inflammatory responses and cytokine synthesis in mast cells, which play important roles in allergic inflammation (Kim et al., 2018). Mast cell–derived pro-inflammatory cytokines, such as TNF-α and IL-6, trigger and sustain mast cell inflammatory responses. TNF-α is a potent pro-inflammatory cytokine with a variety of biological functions associated with inflammation, such as tissue remodeling, recruitment of immune cells, and upregulation of adhesion molecules (Rijnierse et al., 2006). IL-6 is a soluble mediator, produced in response to allergen that causes T-cell activation and IgE production (Tanaka et al., 2014). Therefore, inhibition of inflammatory cytokines is important for reducing allergic inflammation. G.M2 significantly suppressed the gene expression and release of TNF-α and IL-6 in mBMMCs and RBL-2H3 through inhibition of the Akt/NF-κB pathways. In general, these results suggest that G.M2 exhibits anti-inflammatory effects *via* inhibition of transcription factor and these effects are linked to FcεRI-mediated intracellular calcium release.

Elevated intracellular calcium levels are required for exocytosis of mast cell granules (Cohen et al., 2015). This process results from FcεRI-mediated mast cell activation and calcium release (Gilfillan and Tkaczyk, 2006). Intracellular calcium release causes the fusion of preformed granules with the plasma membrane, which results in secretion of inflammatory mediators, such as histamine, β-hexosaminidase, proteases, proteoglycans, and lipid mediators following mast cell degranulation (Cohen et al., 2015). These secreted mediators trigger allergic inflammatory responses. Calcium release is central for driving the degranulation of histamine containing vesicles. Histamine is an important effector mediator released upon stimulation (Krystel-Whittemore et al., 2015). Histamine induces the inflammatory responses *via* vasodilation, increased vascular permeability, hypothermia, and edema (Metcalfe et al., 2009). β-Hexosaminidase is a marker of mast cell degranulation that also triggers allergic inflammatory responses (Fukuishi et al., 2014). In our results, G.M2 inhibited the release of histamine and β-hexosaminidase in a concentration-dependent manner by inhibiting intracellular calcium levels in various types of mast cells. Previous studies showed that inhibition of mast cell degranulation was linked with the suppression of intracellular calcium levels (Bae et al., 2011; Kim et al., 2018). Therefore, we suggest that G.M2 inhibited mast cell degranulation by suppressing FcεRI-mediated intracellular calcium levels.

Anaphylaxis is an immediate-type allergic reaction, generally known as the classical pathway, which involves IgE/FcεRI-mediated mast cell activation and subsequent secretion of inflammatory mediators (Li et al., 2016). IgE-mediated PCA and OVA-induced ASA are well-known models for evaluating the anti-allergic effects of compounds based on local and systemic anaphylaxis (Kim et al., 2017). PCA is an evanescent cutaneous reaction. Intradermal injection of IgE enhances local allergic reactions, such as increased vascular permeability and plasma extravasation. This allows the leakage of DNP-HSA-bound Evans blue dye at the site of IgE injection. As a result, the amount of dye found in the ear is increased by an allergic reaction (Kim et al., 2018). Therefore, G.M2-mediated reduction in leakage of dye and ear thickness in the PCA model suggest an inhibition of allergic reactions. In addition, OVA-sensitized ASA is a generalized allergic reaction manifesting as hypothermia, hypotension, and bronchoconstriction. Repeated administration of OVA sensitization elevates serum histamine and IgE levels and IgE-dependent mast cell activation. The increase in histamine level leads to hypothermia following the OVA challenge (Mathias et al., 2011). According to our findings, oral administration of G.M2 suppressed these reactions. Thus, we suggest that G.M2 inhibits allergic responses by inhibiting mast cell activation.

Mast cell activation has been broadly studied in allergy for identifying new drug candidates (McShane et al., 2015). Anti-histamines, steroids, and anti-IgE drugs have been used as anti-allergic agents for many years (Thurmond et al., 2008). Although these drugs have a rapid effect on mast cell–mediated allergic disease, repeated administration leads to drug resistance and carries side effects, such as drowsiness, dry mouth, vision change, and upset stomach (Sanada et al., 2005). Therefore, identifying novel anti-allergic drugs is important. Our study provides considerable evidence that G.M2 isolated from *S. chinensis* suppressed mast cell–mediated allergic inflammation by inhibiting FcεRI-mediated Lyn and Fyn activation and intracellular calcium levels. Based on these findings, we propose that G.M2 may represent a novel agent for the management of mast cell–mediated allergic disease.

## Data Availability

The raw data supporting the conclusions of this manuscript will be made available by the authors, without undue reservation, to any qualified researcher.

## Ethics Statement

The care and the treatment of the animals were in accordance with the guidelines established by the Public Health Service Policy on the Humane Care and Use of Laboratory Animals and were approved by the Institutional Animal Care and Use Committee of Kyungpook National University (IRB #2016-0001-123).

## Author Contributions

HD carried out the major experiments and drafted the manuscript. SL, E-NK, JC, M-JK, JK, and Y-AC provided support in the study design, reviewed the protocol, and participated to interpret the primary outcome. M-CB, BL, H-SL, and T-YS provided input in the statistical planning and data evaluation. G-SJ and S-HK supervised the research and co-wrote the manuscript.

## Funding

This work was supported by National Research Foundation of Korea grant funded by the Korea government (2014R1A5A2009242, 2017M3A9G8083382, and 2017R1D1A1B03031032), and Korea Health Technology R&D Project of the Korea Health Industry Development Institute (KHIDI, HI18C0308).

## Conflict of Interest Statement

The authors declare that the research was conducted in the absence of any commercial or financial relationships that could be construed as a potential conflict of interest.

## Abbreviations

G.M2, Gomisin M2; mBMMCs, mouse bone marrow derived mast cells; RPMCs, rat peritoneal mast cells; OVA, ovalbumin; PCA, passive cutaneous anaphylaxis; ASA, active systemic anaphylaxis; OPA, *o*-phthaldialdehyde.
