# Supplementary material for: Gomisin M2 Inhibits Mast Cell-Mediated Allergic Inflammation via Attenuation of FcεRI-Mediated Lyn and Fyn Activation and Intracellular Calcium Levels
Source: Front Pharmacol. 2019 Aug 2;10:869. doi: 10.3389/fphar.2019.00869 (PMC6688163; doi:10.3389/fphar.2019.00869)
Supplement: Supplementary file 1 [file DataSheet_1.docx]

**Gomisin M2 inhibits mast cell-mediated allergic inflammation *via* attenuation of FcεRI-mediated Lyn and Fyn activation and intracellular calcium levels**

**Supplementary Figure S1**

**Figure S1. Effects of schisandrin, gomisin A, and G.M2 in mast cell degranulation.**

Anti-DNP (50 ng/mL) sensitized mBMMCs (5 × 10^5^ cells/well in 12-well plates) were pre-treated with or without schisandrin (Schi), gomisin A (G.A), and G.M2 or Dexa for 1 h and then challenged with DNP-HSA (100 ng/mL) for 30 min. (A) Histamine levels were detected with fluorescent plate reader. (B) The level of β-hexosaminidase was measured using β-hexosaminidase substrate buffer. Each data presented as a graph represents the means ± SEM of three independent experiments. **p* < 0.05, compared with the DNP-HSA challenged group. Dexa: dexamethasone.

**Supplementary Figure S2**

**
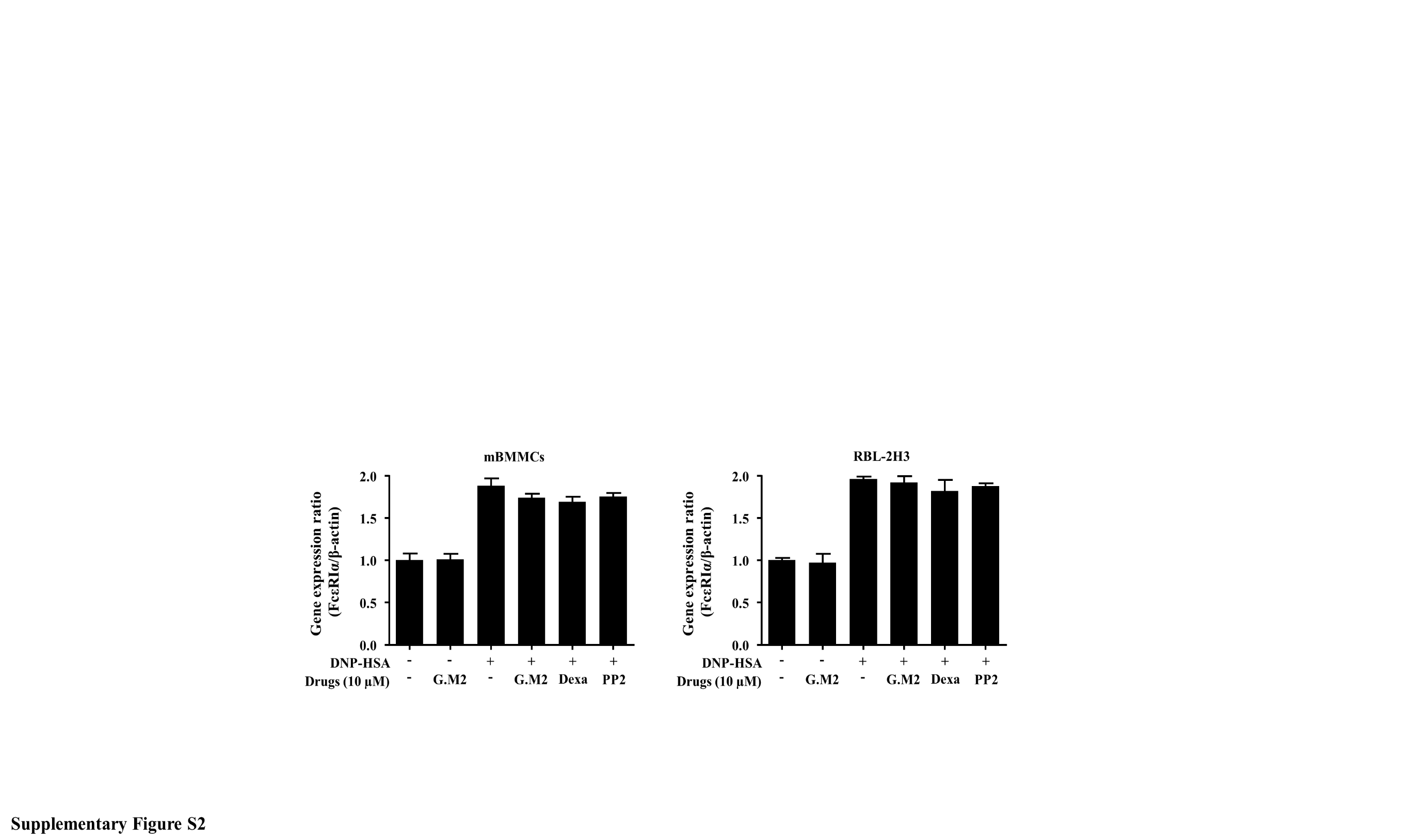
**

**Figure S2. Effects of G.M2 in gene expression of FcεRIα.**

Anti-DNP IgE sensitized mBMMCs and RBL-2H3 (5 × 10^5^ cells/well) were pre-treated with G.M2, Dexa or PP2 for 1 h and stimulated with DNP-HSA for 6 h. The gene expression of FcεRIα was determined by qPCR. Each data presented as a graph represents the means ± SEM of three independent experiments. Dexa: dexamethasone.

**Supplementary Figure S3.**

**
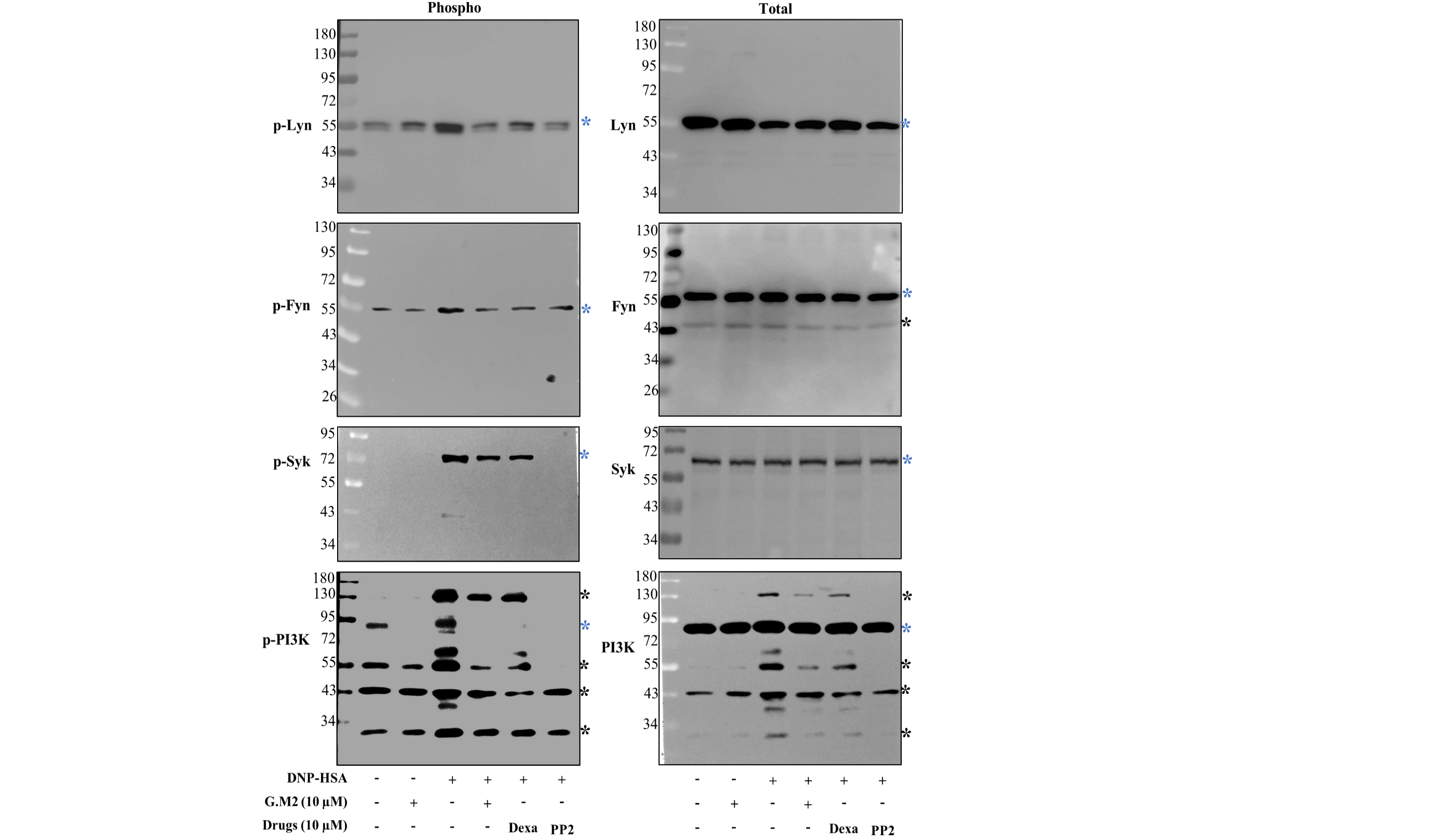
**

**
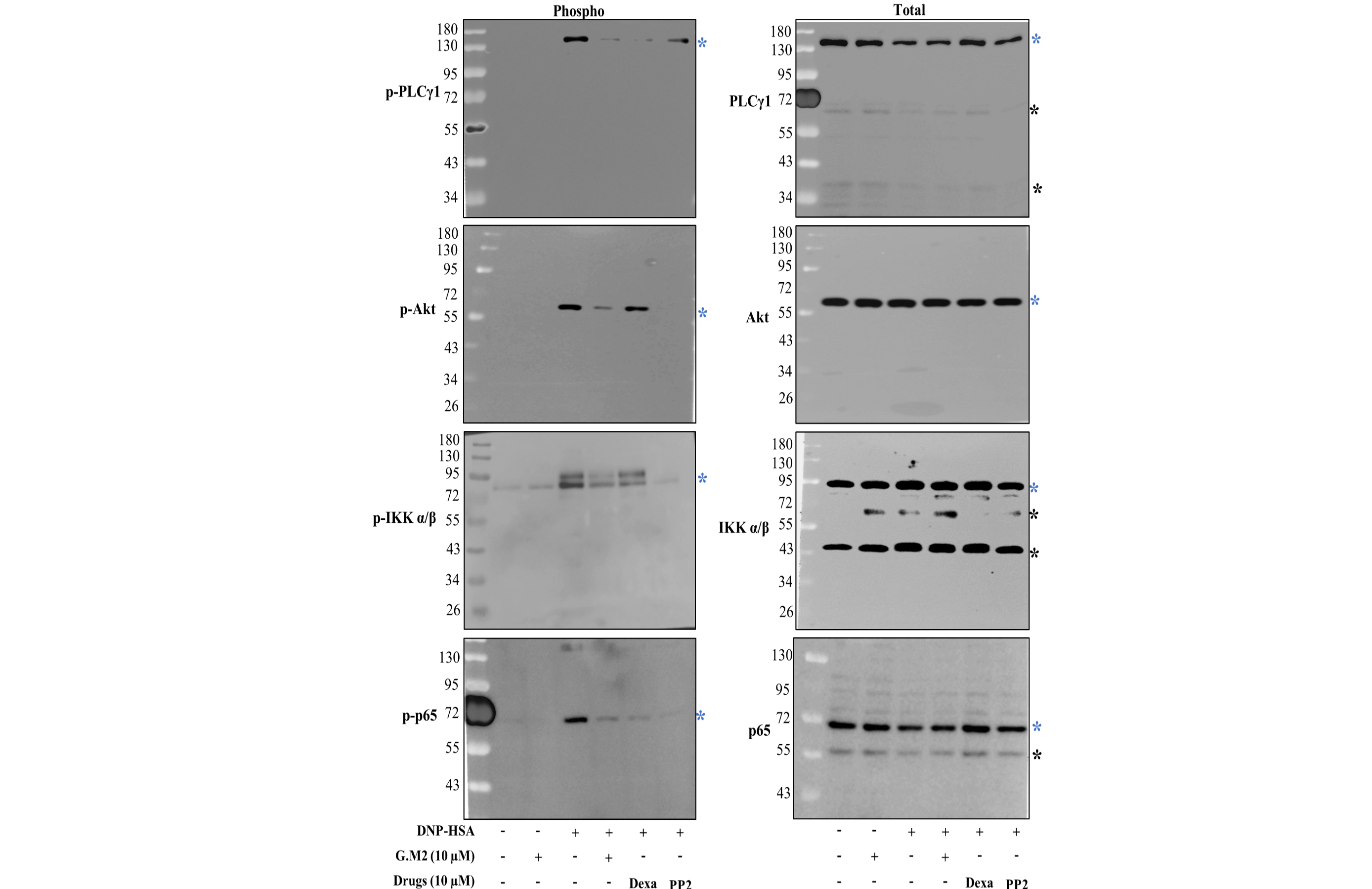
**

**
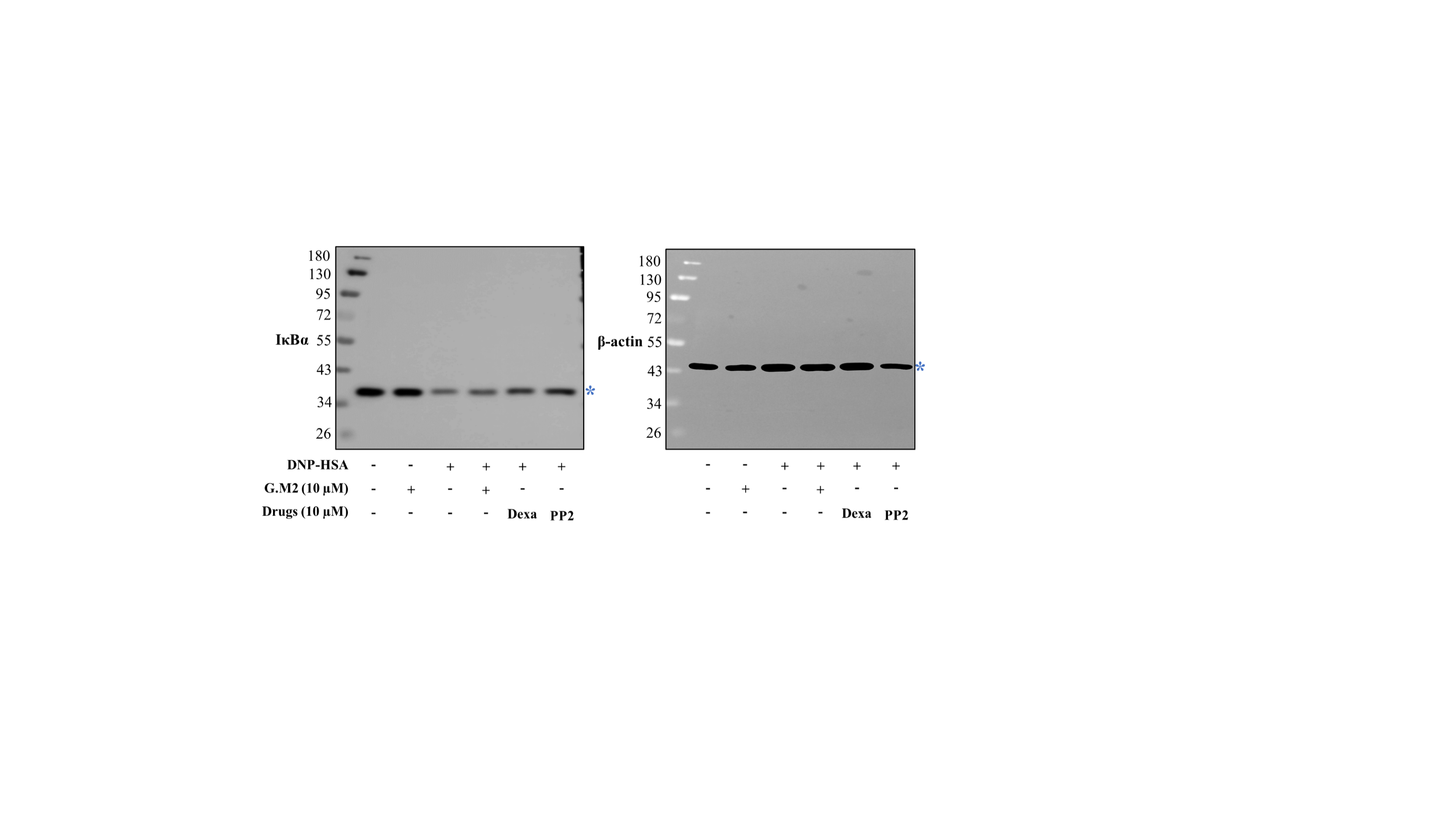
**

**Figure S3. Full-length Western blot corresponding to Figure 4.**

Asterisk (black) denotes non-specific bands and asterisk (blue) denotes the target band, which are used in manuscript file.

**Supplementary Table S1. Primers used in qPCR analysis.**

| Name | Forward (5*^′^*-3*^′^*) | Reverse (5*^′^*-3*^′^*) |
| --- | --- | --- |
| mβ-actin | ACCCTAAGGCCAACCGTGAA | ATGGCGTGAGGGAGAGCATAG |
| mTNF-α | GGCAGGTCTACTTTGGAGTCATTGC | ACATTCGAGGCTCCAGTGAATTCGG |
| mIL-6 | CCACTTCACAAGTCGGAGGCTTA | GCAAGTGCATCATCGTTGTTCATAC |
| mFcεRIα | AACTGGAATGTCCGCAAGGT | GAATCGCCACCAACAATGGG |
| rβ-actin | GAAGCTGTGCTATGTTGCCCTAGA | GTACTCCTGCTTGCTGATCCACAT |
| rTNF-α | TCCCAA[ATG](https://www.sciencedirect.com/topics/pharmacology-toxicology-and-pharmaceutical-science/thymocyte-antibody)GGCTCCCTCTC | AAATGGCAAACCGGCTGACG |
| rIL-6 | CAGATTGTT[TTC](https://www.sciencedirect.com/topics/pharmacology-toxicology-and-pharmaceutical-science/triphenyltetrazolium)TGACAGTG | CAGGGAGATCTTGGAAATGA |
| rFcεRIα | ATCGTCACATTGGTCATTG | CACGTCAGCAGAAGATTGGA |

**Antibodies used in Western blot analysis.**

1. Phospho-Lyn (Cell Signaling Technology, #2731, rabbit polyclonal, 1:1000)
2. Phospho-Fyn (Abcam, ab182661, rabbit polyclonal, 1:1000)
3. Phospho-Syk (Cell Signaling Technology #2711, rabbit polyclonal, 1:1000)
4. Phospho-Akt (Cell Signaling Technology, #9271, rabbit polyclonal, 1:1000)
5. Phospho-PI3K (Cell Signaling Technology, #4228, rabbit polyclonal, 1:1000)
6. Phospho-PLCγ (Cell Signaling Technology, #2821, rabbit polyclonal, 1:1000)
7. Phospho-p65 NF-κB (Cell Signaling Technology, #3631, rabbit polyclonal, 1:1000)
8. Lyn (Cell Signaling Technology, #2732, rabbit polyclonal, 1:1000)
9. Fyn (Abcam, ab125016, rabbit polyclonal, 1:1000)
10. Syk (Cell Signaling Technology, #2712, rabbit polyclonal, 1:1000)
11. Akt (Cell Signaling Technology, #9272, rabbit polyclonal, 1:1000)
12. PI3K (Cell Signaling Technology, #4292, rabbit polyclonal, 1:1000)
13. PLCγ (Cell Signaling Technology, #2822, rabbit polyclonal, 1:1000)
14. p65 NF-κB (Santa Cruz, sc-109, rabbit polyclonal, 1:1000)
15. IκBα (Santa Cruz, sc-371, rabbit polyclonal, 1:1000)
16. β-actin (Santa Cruz, sc-8432, mouse monoclonal, 1:1000)
